# Gandi Capsule Improved Podocyte Lipid Metabolism of Diabetic Nephropathy Mice through SIRT1/AMPK/HNF4A Pathway

**DOI:** 10.1155/2022/6275505

**Published:** 2022-04-18

**Authors:** Ying Zhang, Huijuan Yao, Chao Li, Wei Sun, Xiaofei Chen, Yan Cao, Yue Liu, Yan Liu, Jihui Chen, Jia Qi, Qiqiang Zhang, Hai Zhang, Ajing Xu, Jian Zhang

**Affiliations:** ^1^Department of Clinical Pharmacy, Xinhua Hospital, Shanghai Jiao Tong University School of Medicine, Shanghai 200092, China; ^2^Innovation Research Institute of Traditional Chinese Medicine, Shanghai University of Traditional Chinese Medicine, Shanghai 201203, China; ^3^School of Pharmacy, Second Military Medical University, Shanghai 200433, China; ^4^Department of Pharmacy, Shanghai First Maternity and Infant Hospital, Tongji University School of Medicine, Shanghai 201204, China

## Abstract

Podocyte lipid accumulation is a potential therapeutic target for diabetic nephropathy (DN). This study was aimed at clarifying the mechanism of Gandi capsule (GDC) ameliorating DN by regulating the lipid metabolism of podocytes. Network pharmacology methods were performed to screen the key molecules and potential targets of GDC for constructing the molecular-protein interaction network of GDC and conducting signal pathway enrichment analysis. GDC was predicted to ameliorate DN through SIRT1/AMPK/HNF4A pathway. Our results showed that GDC improved renal function in db/db mice. Besides, GDC exhibited effectiveness in relieving kidney tissue damage and renal lipid accumulation in db/db mice, and same effects were present in GDC-active ingredient baicalin. We further proved the new role of HNF4A in the lipid metabolism of DN mediated by SIRT1 and AMPK signaling pathways. The results suggested decreased expression of SIRT1 and p-AMPK*α* in the kidney tissue and increased expression of HNF4A of db/db mice compared with the control group. GDC and baicalin could reverse these expression changes. Furthermore, similar expression changes were observed in the murine podocyte cell line (MPC-5) treated with different concentrations of GDC and baicalin. Our research suggested that GDC and its active ingredient baicalin could alleviate the abnormal lipid metabolism in the kidney of db/db mice and might exert renal protection through the SIRT1/AMPK/HNF4A pathway.

## 1. Introduction

The main clinical manifestations of diabetic nephropathy (DN) are proteinuria, progressive kidney damage, hypertension, and severe renal failure, leading to the end-stage renal disease (ESRD) [1, 2]. The prevalence of diabetes mellitus worldwide is increasing with the growing rate of obesity, which to some extent results in the global burden of DN [3]. Adipokines and ectopic lipid accumulation in the kidney stimulate insulin resistance and oxidative stress of podocytes to deal with the pressure of renal hyperfiltration. Altered fatty acid and cholesterol metabolism are considered as a key approach for renal lipid accumulation, inflammation, oxidative stress, and fibrosis. Novel therapies targeted at lipid metabolism, including SREBP antagonists, PPAR*α* agonists, and LXR agonists, hold therapeutic promise [4].

In recent years, there has been a great breakthrough in traditional Chinese medicine (TCM) therapies for DN as TCM with different ingredients exhibited multitargets to the remission of DN. Gandi capsule (GDC) has been clinically proven to reduce urinary microalbumin in patients with DN effectively [5, 6]. Our previous studies demonstrated that lipid metabolism pathway was one of the main metabolic pathways of GDC in protecting patients with DN [7]. Natural active substance baicalin has been proved to improve lipid accumulation by anti-inflammatory and antioxidation effects [8]. Baicalin ameliorated hepatic insulin resistance and gluconeogenic activity through inhibition of p38 MAPK/PGC-1*α* pathway [9]. In addition, baicalin regulated SIRT1/STAT3 pathway and restrained excessive hepatic glucose production [8]. Baicalin was found to be effectively bound with HNF4A and SIRT1 through network pharmacology and molecular docking analysis [10], which suggested the key role of baicalin in glucose and lipid metabolism. However, its mechanism of reducing renal lipid accumulation remains unclear. As the active substance of GDC, the effect of baicalin on reducing lipid metabolism reflects the mechanism of Gandi capsule in treating DN.

Ameliorating lipotoxicity is the novel mechanistic insight in DN [11]. The excessive lipid accumulation activates the lipogenic and glycogenic cell signaling pathways, resulting in podocyte dysfunction [12, 13]. Podocyte is highly differentiated epithelial cells attached to the glomerular basement membrane, and it forms the outermost layer of the glomerular filtration barrier, which is essential for the glomerular filtration barrier and implicated in proteinuria of DN [14]. Lipotoxicity leads to the loss of podocytes in DN, which also directly affects the efficacy of statins targeting on cholesterol synthesis in the clinical treatment of DN [15]. Therefore, relieving renal lipotoxicity is a potential therapeutic approach to delay the progression of DN.

Therefore, this study was conducted to clarify the mechanism of GDC ameliorating DN by regulating the lipid metabolism of podocytes. We found that GDC treatment ameliorated renal injury and proteinuria as well as significantly decreased triglyceride level in db/db mice. Importantly, GDC was found to alleviate lipid accumulation in MPC-5 through SIRT1/AMPK/HNF4A pathway. Similar results were also shown in baicalin-treated db/db mice and MPC-5, which suggested the potential attractive and effective therapeutics of GDC in DN.

## 2. Materials and Methods

### 2.1. Herbal Formulation

Eight Chinese herbs in the GDC formula (Table 1) were purchased from Shanghai Traditional Chinese Medicine Pharmaceutical Technology Co., Ltd. (Shanghai, China). The content determination and identification tests (such as TLC and HPLC) were carried out according to the Pharmacopeia of China (2020). The preparation of GDC extract was carried out as the following description. Prescription medicinal materials were weighed and dissolved in water to extract 2 times, 1 hour each time. Water was added 10 times and 8 times, respectively, at first and second 1 hour. Then, the first and second decoctions were collected and filtered, and the extract was concentrated to a relative density of about 1.20 ± 0.02 kg/m^3^ and centrifuged. The supernatant was concentrated into concrete, and an appropriate amount of excipients were added to make GDC mixture. The doses of GDC for mice were 1.5 g·kg^−1^day^−1^ and 3 g·kg^−1^day^−1^. The HPLC chromatograms and TLC chromatograms were used to detect marker constituents of GDC (supplement).

(1) $$\begin{array}{c} \text{Yield}=735.5\text{ g dry extract}/3116.7\text{ g the total materials}=23.6\%\left(\text{W}/\text{W}\right) \end{array}$$

### 2.2. Animals and Drug Administration

Eight-week-old C57BL/Ksj db/m normal and db/db diabetic mice were purchased from Shanghai Model Organisms Center, Inc. (certificate number: SCXK [Jiangsu] 2018-0008, China). All animals were kept in the barrier-level animal room at constant room temperature 22 ± 2°C and controlled humidity (40%-70%) with a light/dark cycle of 12 hours. All the animals were ad libitum allowed to access the standard diet (mouse crackers) and water throughout the day.

Research showed that fasted blood glucose level higher than 16.7 mmol/L with moderate to severe albuminuria was model criteria for diabetic nephropathy mice [16–18]. We found that most of the 11-week-old db/db mice could meet the criteria. At 11 weeks old, db/db mice were randomly divided into five groups (*n* = 6), db/db group (model control), db/db+1.5 g·kg^−1^day^−1^ Gandi capsule group (db/db+GDC^a^), db/db+3.0 g·kg^−1^day^−1^ Gandi capsule group (db/db+GDC^b^), db/db+20 mg·kg^−1^day^−1^ valsartan group, and db/db+100 mg·kg^−1^day^−1^ baicalin group. For the substances administration, GDC and valsartan dissolved in pure water and baicalin dissolved in 0.5% CMC-Na were delivered into the stomach by a 20 G curved gavage needle once a day. The mice in the control group were administered with the corresponding vehicle, pure water or 0.5% CMC-Na. This study was approved by the Institutional Animal Care and Use Committee (IACUC) of Shanghai University of Traditional Chinese Medicine (IACUC-SR-2110). After 8 weeks of treatment, the mice were anesthetized with isoflurane, and then, the blood sample was collected from the inferior vena cava for subsequent biochemical analysis, and the kidney samples were harvested for histopathological analysis.

### 2.3. Cell Culture and Treatment

The conditionally immortalized murine podocyte cells were cultured in RPMI 1640 (Mediatech, Herndon, VA, USA) containing 10% fetal bovine serum (FBS, Hyclone, Logan, UT, USA), 20 U/mL mouse recombinant interferon-*γ* (IFN-*γ*, Pepro Tech, Cranbury, NJ, USA), and 100 U/mL penicillin plus 0.1 mg/mL streptomycin at 5% CO_2_ humidified in 33°C growth-permissive conditions or in 37°C growth-restrictive conditions without IFN-*γ* for 5-7 days to induce differentiation before treatment. All the cells were synchronized via starvation in serum-free medium for 6 hours and then cultured for 24 hours with normal glucose (NG, 5.5 mmol/L glucose), mannitol as the osmotic pressure control (M, 5.5 mmol/L glucose +24.5 mmol/L mannitol), and high glucose (HG, 30 mmol/L glucose). GDC and baicalin were pretreated 1 hour before high glucose treatment.

### 2.4. Cell Counting Kit-8 Assay

MPC-5 was treated with GDC and baicalin by different concentration for 24, 48, and 72 h. Cell viability was measured at 450 nm wavelength by a microplate reader (Synergy LX Multi-Mode Reader, USA) under the instructions of Cell Counting Kit-8 (CCK-8, Yeasen, Shanghai, China).

### 2.5. Biochemistry Measurements

Fasting blood glucose was measured by a stable nontuning blood glucose meter (Sinocare, China). Blood glucose (GLU), albuminuria (mg/24 h), serum creatinine (CREA), blood urea nitrogen (BUN), serum triglyceride (TG), total cholesterol (TC), low-density lipoprotein-cholesterol >(LDL-C), and high-density lipoprotein-cholesterol (HDL-C) levels were measured using an automatic analyzer (HITACHI 7080, Japan).

### 2.6. Measurement of Triglyceride and Cholesterol Levels in the Kidney

Renal tissue was accurately weighed, homogenized, and centrifugated. The supernatants were taken for triglyceride and cholesterol testing using an enzyme reaction kit (Nanjing, China), and the results were normalized to the protein concentration.

### 2.7. Hematoxylin and Eosin (HE), Oil Red O, and Immunohistochemistry Staining

Renal tissue fixed with formalin (10%) was sectioned at 5 mm for hematoxylin and eosin (H&E) staining. The sections of frozen renal tissue and cells were for oil red O staining. The paraffin sections for immunohistochemistry were incubated with anti-podocin/NPHS2 (Novus, Littleton, CO, USA) overnight at 4°C. After being incubated with HRP-conjugated secondary antibody, the sections were counterstained with hematoxylin and developed with DAB (3,3′-diaminodbenzidine) and then covered with coverslips and natural resin.

### 2.8. The Construction and Analysis of Compound-Target-Pathway Network

To analyze the interaction of the main active compounds of GDC, protein targets, and pathway in diabetic nephropathy, a network diagram was constructed by Cytoscape 3.6.0 and ClueGo 3.6.0 analytical software. The compound protein network was constructed by the interaction of the candidate molecule with the protein targets. The disease-pathway network was built through the interaction of the diabetic nephropathy and the pathways (Zhang et al., 2020). The information was collected from TCMSP (https://old.tcmsp-e.com/tcmsp.php).

### 2.9. Western Blot Analysis

Renal tissue and cultured MCP-5 were homogenized and lysed in RIPA buffer (Biosharp, China). Protein extracts were subjected to SDS-PAGE and transferred to nitrocellulose membranes. The primary antibodies were as follows: rabbit monoclonal podocin/NPHS2 antibody (Novus, Littleton, CO, USA, 1 : 1000), rabbit SIRT1 (Proteintech, Rosemont, IL, USA, 1 : 1000), rabbit HNF4A (ABclonal Technology, Woburn, MA, USA, 1 : 1000), rabbit p-AMPK (Cell Signaling Technology, Danvers, MA, USA, 1 : 1000), rabbit AMPK (Proteintech, Rosemont, IL, USA, 1 : 1000), and rabbit *β*-actin (Cell Signaling Technology, Danvers, MA, USA, 1 : 5000). The membranes were incubated with anti-rabbit IgG (H+L) (Cell Signaling Technology, Danvers, MA, USA, 1 : 10000) and then detected using Odyssey® Sa Infrared Imaging System (LI-COR Biosciences, United States). Quantification was performed by ImageJ (version 1.8.0).

### 2.10. Statistical Analysis

Quantitative data were shown as the means ± standard deviation. Comparisons among groups were performed by a one-way analysis of variance (ANOVA) coupled with Tukey's post hoc test, and GraphPad Prism (version 8.0) was used for the analysis. Statistically significance was defined as *P* < 0.05.

## 3. Results

### 3.1. Active Components of Gandi Capsule Improve Diabetic Nephropathy through Multiple Targets

**Figure 1:**
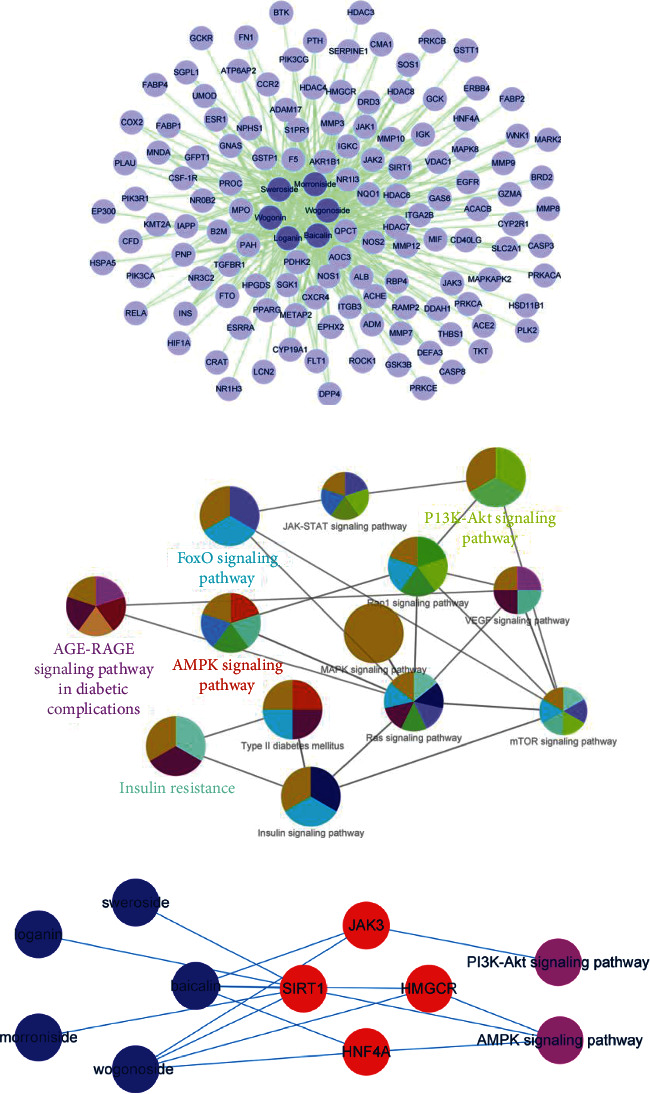
Screening of key molecules and target in Gandi capsule by network pharmacological analysis. (a) Molecule-protein interaction network analysis constructed by Cytoscape software. (b) Pathway enrichment analysis and core pathway screening by ClueGo software. (c) Core molecule-protein-pathway network.

Network pharmacology approach is applied to map the unexplored target of natural products, thereby establishing a systematic mean to explore the unknown target proteins related to various complex diseases [19]. The 126 proteins associated with diabetic nephropathy and the 6 active ingredients contained in the GDC were selected by molecule-protein interaction network analysis (Figure 1(a)). Through pathway enrichment analysis of the screened proteins, the AMPK pathway and PI3K-Akt pathway were closely related to diabetic nephropathy (Figure 1(b)). Analyzing the representative pathways and targets, the relationship of compound-target pathway was represented to establish the core molecule-protein-pathway network (Figure 1(c)). We found that baicalin was closely related to potential targets, HNF4A and SIRT1, and jointly pointed to AMPK pathway (Figure 1(c)).

### 3.2. GDC and Baicalin Treatment Improved Renal Function and Ameliorated Dyslipidemia in db/db Mice

**Figure 2:**
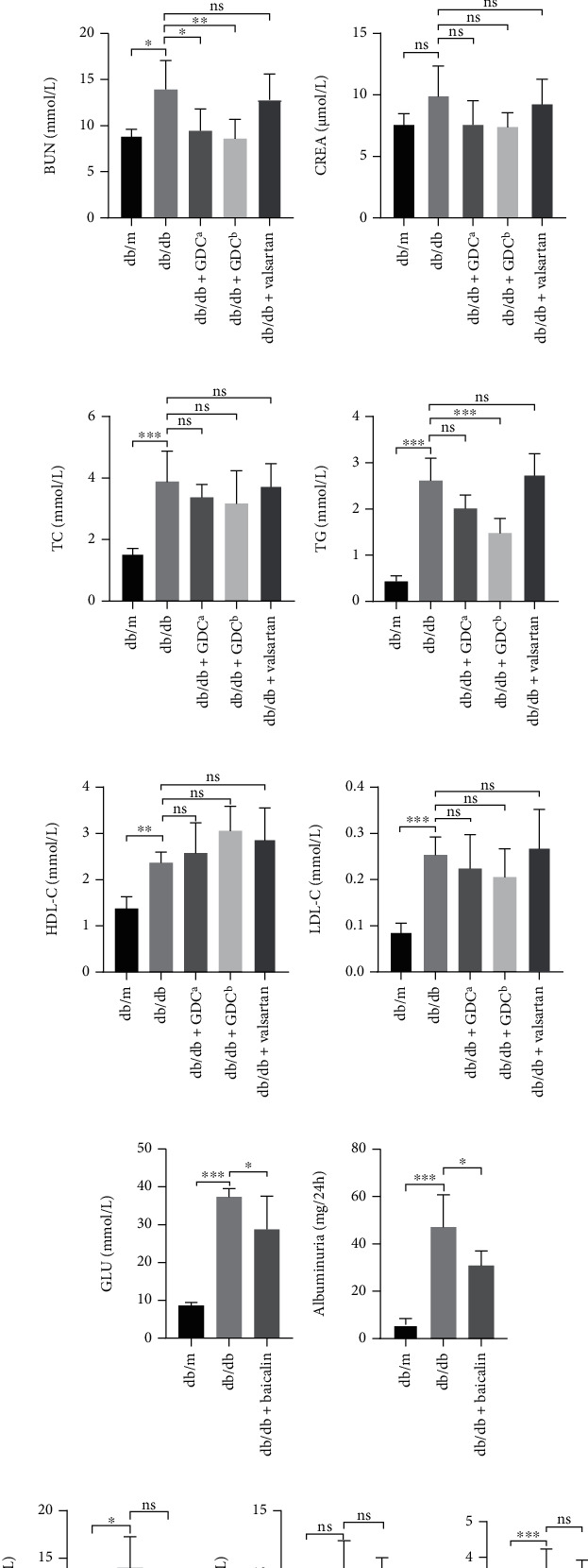
GDC and baicalin ameliorated renal function and disorder of renal lipid metabolism in 18-week-old db/db mice. (a and b) Administration of (a) GDC and (b) baicalin reduced the body weight of diabetic nephropathy mice. (c) Serum GLU, (d) albuminuria, (e) BUN, (f) CREA, (g) serum TC, (h) TG, (i) HDL-C, and (j) LDL-C of mice treated with GDC or valsartan were measured at the 18th week. (k) Serum GLU, (l) albuminuria, (m) BUN, (n) CREA, (o) Serum TC, (p) TG, (q) HDL-C, and (r) LDL-C of mice treated with baicalin were measured at the 18th week. ^a^1.5 g·kg^−1^day^−1^, ^b^3.0 g·kg^−1^day^−1^, ∗*P* < 0.05, ∗∗*P* < 0.01, and ∗∗∗*P* < 0.001.

We investigated the efficacy of GDC and baicalin by establishing an animal model of diabetic nephropathy. After 8 weeks, the weight of db/db mice was increased compared to db/m mice and decreased significantly after GDC and baicalin treatment (Figures 2(a) and 2(b)**)**. The levels of blood glucose, albuminuria, and BUN were increased in db/db group compared with db/m group, and albuminuria level was significantly decreased in GDC group compared to the db/db group; however, the CREA was not significant among these groups (Figures 2(c)–2(f)). Serum levels of TG, TC, HDL-C, and LDL-C were raised in db/db group compared with db/m group, and TG was decreased in GDC group compared with db/db group (Figure 2(g)–2(j)). We found that the levels of blood glucose and albuminuria were reduced, and BUN and CREA were not affected by the baicalin group compared with db/db group (Figures 2(k)–2(n)). Serum concentrations of TG, TC, HDL-C, and LDL-C were remarkably raised in db/db group, of which TG was decreased in baicalin group compared with db/db group (Figures 2(o)–2(r)). Therefore, we concluded that GDC and baicalin could reduce albuminuria and triglyceride levels, while baicalin could reduce both glucose and triglyceride in diabetic nephropathy mice.

### 3.3. GDC and Baicalin Treatment Alleviated Histological Damage and Lipid Accumulation in db/db Mice

**Figure 3:**
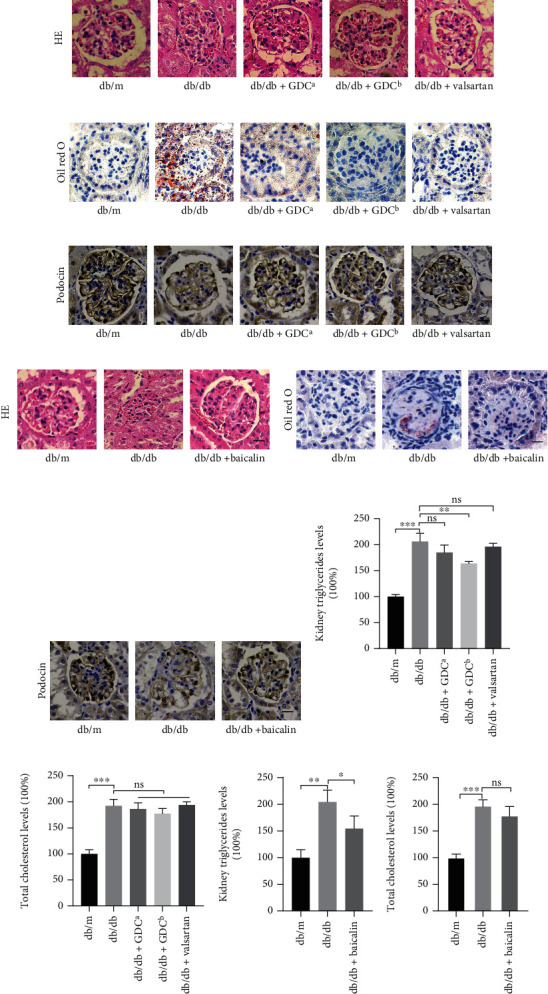
Renal histologic changes and lipid accumulation in 18-week-old db/db mice treated with GDC or baicalin. (a) H&E, (b) Oil red O stain, and (c) immunohistochemistry staining for podocin were presented about diabetic nephropathy mice treated by DGC. Scar bar, 100 *μ*m. Valsartan group as a positive control. (d) H&E, (e) Oil red O stain, and (f) immunohistochemistry staining for podocin were presented about diabetic nephropathy mice treated by baicalin. Scar bar, 100 *μ*m. (g–j) Triglyceride and cholesterol levels were analyzed by colorimetric assay of renal tissue. ∗*P* < 0.05, ∗∗*P* < 0.01, and ∗∗∗*P* < 0.001.

To assess the effect of GDC and baicalin on renal histology in db/db mice, renal damages of db/db group were manifested by the disappearing of the glomerular basal membrane and the increasing mesangial matrix of glomerular by HE staining. The kidney in GDC group showed loss of its normal structure with mild lesions (Figure 3(a)). Oil red O staining revealed that db/db group had mass lipid droplets in the glomerulus, and GDC group significantly reduced the lipid droplet accumulation compared with the db/db group (Figure 3(b)). Immune staining indicated that the levels of podocin decreased markedly in renal tissue in db/db group and increased after GDC treatment (Figure 3(c)). Similarly, baicalin significantly ameliorated the mesangial matrix increases of glomerular (Figure 3(d)), decreased the staining area of oil red O (Figure 3(e)), and restored the levels of podocin in renal tissue (Figure 3(f)) compared with the db/db group. Colorimetric assay also indicated that GDC and baicalin treatment decreased the renal triglyceride level and had no effect on the total cholesterol level in the renal tissues compared with db/db group (Figures 3(g)–3(j)). Consequently, both GDC and baicalin treatment alleviated histological damage and lipid accumulation in db/db mice.

### 3.4. GDC and Baicalin Increased AMPK Phosphorylation and SIRT1 Expression and Decreased HNF4A Expression in db/db Mice

**Figure 4:**
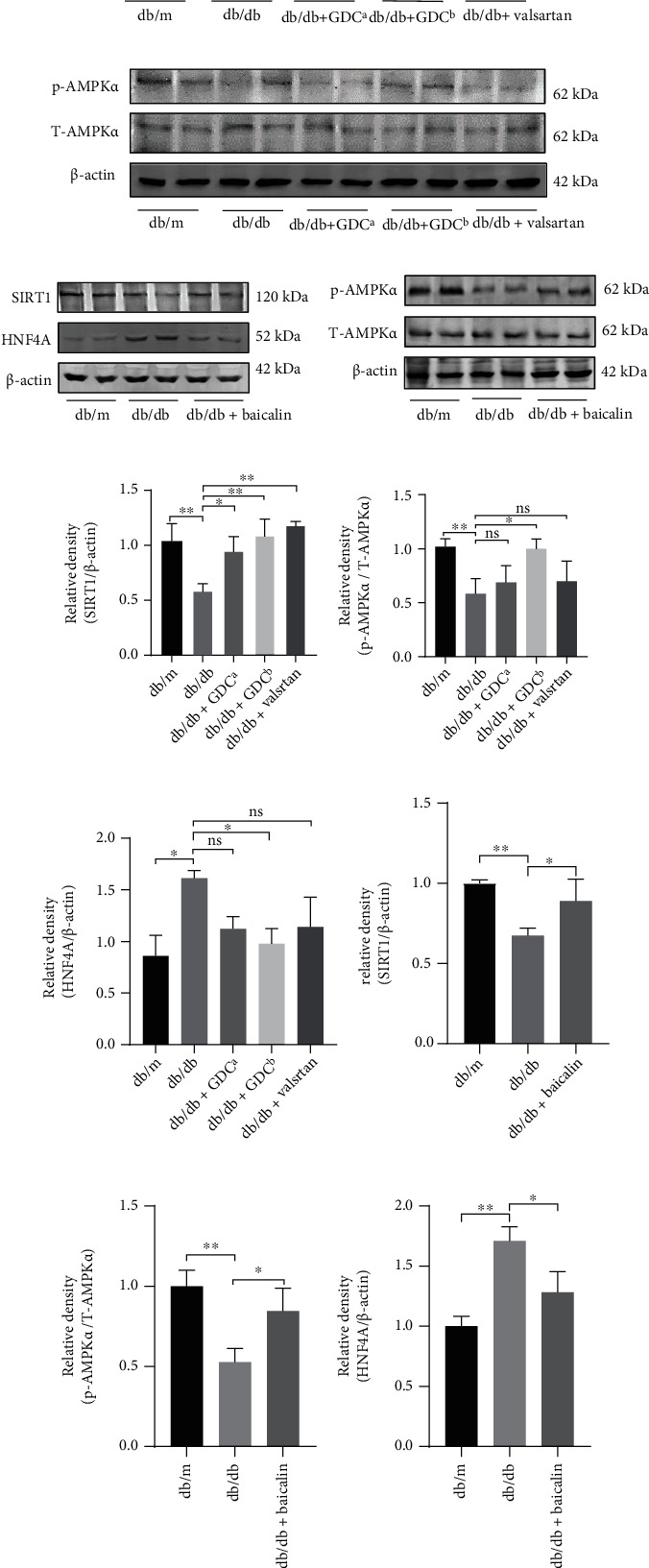
The expression of SIRT1, p-AMPK*α*/total AMPK*α*, and HNF4A in mice renal tissue treated with GDC, valsartan, and baicalin. (a and b) The levels of SIRT1, p-AMPK*α*, T-AMPK*α*, and HNF4A in mice renal tissue treated with GDC were analyzed by western blot. (c and d) The levels of SIRT1, p-AMPK*α*, T-AMPK*α*, and HNF4A in mice renal tissue treated with baicalin were analyzed by western blot. (e–g) Quantitative analyses of western blot by GDC treatment: SIRT1/*β*-actin; p-AMPK*α*/total AMPK*α*; and HNF4A/*β*-actin. (h–j) Quantitative analyses of western blot by baicalin treatment: SIRT1/*β*-actin; p-AMPK*α*/total AMPK*α*; and HNF4A/*β*-actin. ∗*P* < 0.05, ∗∗*P* < 0.01, and ∗∗∗*P* < 0.001.

Western blot in vivo was used to test whether GDC and baicalin improved lipid accumulation in diabetic nephropathy mice through SIRT1/AMPK/HNF4A (Figures 4(a)–4(d)). The results showed that the ratio of p-AMPK*α* to AMPK*α* and the expression of SIRT1 were decreased, while the expression of HNF4A was increased in renal tissue of model group compared with the db/m group. Compared with the db/db group, p-AMPK*α*/AMPK*α* ratio and SIRT1 expression were elevated, and HNF4A expression was apparently downregulated by GDC treatment (Figures 4(a) and 4(b)), and the same results were found in baicalin group (Figures 4(c) and 4(d)). Quantitative analyses of the results from the blot were shown below (Figures 4(e)-4(j)). Consequently, the results revealed that GDC and baicalin treatment might ameliorate renal lipid accumulation through the SIRT1/AMPK/HNF4A pathway.

### 3.5. GDC and Baicalin Alleviated Steatosis in HG-Stimulated Murine Podocyte Cells

**Figure 5:**
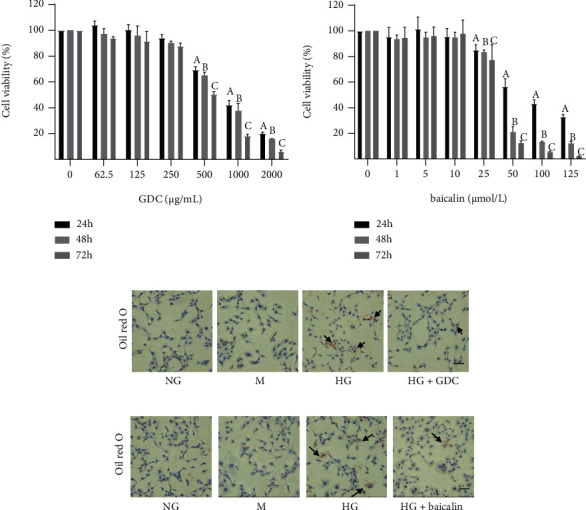
GDC and baicalin alleviated steatosis in HG-stimulated murine podocyte cells. (a) Proliferation of MPC-5 treated by GDC was tested by using CCK-8. (b) Proliferation of MPC-5 treated by baicalin was tested by using CCK-8. (c) The images were oil red O staining of MPC-5 treated by GDC (bar = 50 *μ*m). (d) The images were oil red O staining of MPC-5 treated by baicalin (bar = 50 *μ*m). ^A^*P* < 0.05 compared to MPC-5 cultured under normal glucose for 24 h; ^B^*P* < 0.05 compared to MPC-5 cultured under normal glucose for 48 h; ^C^*P* < 0.05 compared to MPC-5 cultured under normal glucose for 72 h.

To confirm the protective effects of GDC and baicalin against the lipid metabolism imbalance in podocytes, we evaluated the inhibiting effects of GDC and baicalin on intracellular lipid accumulation in MPC-5 treated by high glucose. The CCK-8 results indicated that GDC level below 250 *μ*g/mL (Figure 5(a)) and baicalin level below 10 *μ*mol/L (Figure 5(b)) had no significant effect on the survival rate of MPC-5.

Next, we presented lipid metabolism specifically in MPC-5 by oil red O staining. The cells were cultured under NG, M, HG, HG+125 *μ*g/mL GDC, and HG+12 *μ*mol/L baicalin for 24 hours. Lipid droplet accumulation was higher in HG group compared with the normal group while mild in GDC and baicalin treatment group (Figures 5(c) and 5(d)). Therefore, our results indicated that GDC and baicalin treatment reduced the lipid accumulation in renal podocytes.

### 3.6. GDC and Baicalin Alleviated Lipid Accumulation in Podocytes by Enhancing the Expression of AMPK and SIRT1 and Decreased the Expression of HNF4A in HG-Stimulated MPC-5

**Figure 6:**
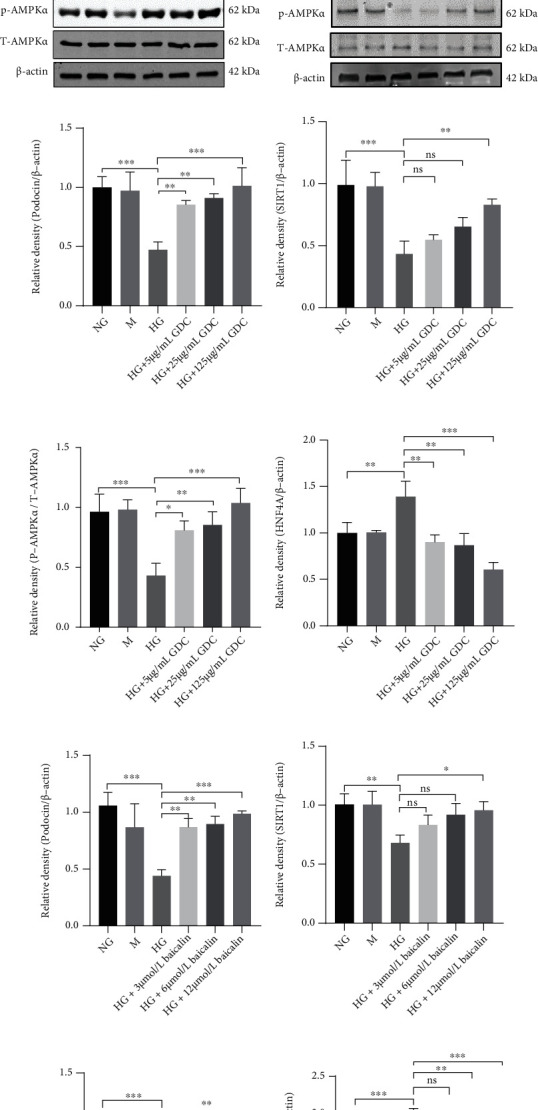
The expressions of podocin, SIRT1, p-AMPK*α*, total AMPK*α*, and HNF4A in MPC-5 after treatment with different concentration of GDC (5, 25, and 125 *μ*g/mL) and baicalin (3, 6, and 12 *μ*mol/L) for 24 hours. (a–f) The protein levels of podocin, SIRT1, p-AMPK*α*, total AMPK*α*, and HNF4A were analyzed in MPC-5 treated with (a, c, and e) GDC or (b, d, and f) baicalin by western blot. (g–j) Quantitative analyses of the GDC results from the blot were shown: (g) podocin/*β*-actin; (h) SIRT1/*β*-actin; (i) p-AMPK*α*/total AMPK*α*; and (j) HNF4A/*β*-actin. (k–n) Quantitative analyses of the baicalin results from the blot were shown: (k) podocin/*β*-actin; (l) SIRT1/*β*-actin; (m) p-AMPK*α*/total AMPK*α*; and (n) HNF4A/*β*-actin. ∗*P* < 0.05, ∗∗*P* < 0.01, and ∗∗∗*P* < 0.001.

It was proved that inhibition of SIRT1-mediated AMPK signaling led to lipid accumulation in HG-induced podocytes [12], and we verified this pathway by western blot in vitro (Figures 6(a)–6(f)). Podocin is one of the protective proteins of renal podocytes. The expression of podocin decreased in HG-induced murine podocyte cells, while GDC and baicalin treatment significantly restored the expression of podocin in our results (Figures 6(a) and 6(b)). In addition, the expression of SIRT1 was upregulated compared with the HG group, while the expression of HNF4A was significantly downregulated in MPC-5 exposed to GDC and baicalin (Figures 6(c) and 6(d)). Furthermore, the decreased expression of p-AMPK in HG-induced murine podocyte cells was significantly upregulated after the treatment of GDC and baicalin (Figures 6(e) and 6(f)). Quantitative analyses of the results from the blot were shown below (Figures 6(g)–6(n)). Therefore, GDC and baicalin could protect renal podocytes injury by restoring the levels of podocin. In addition, GDC and baicalin might alleviate lipid accumulation in podocytes by enhancing the expression of SIRT1 and AMPK and decreasing the expression of HNF4A in HG-stimulated MPC-5.

## 4. Discussion

Diabetes mellitus frequently coexists with obesity, resulting in lipid accumulation in kidney [20], which is called the notion of “fatty kidney disease.” Unsatisfactory glycemic management and renal lipotoxicity have been generally considered as the main contributors to DN progression [12, 13, 21]. Therefore, kidney lipid homeostasis has gained increasing attention in recent years [11].

In our data, abnormal renal lipid metabolism was shown to be present in the kidneys of diabetic nephropathy mice and HG-induced renal podocytes. At the same time, GDC was found to lower serum triglyceride and reduce the lipid droplet accumulation in the kidney and podocytes. However, the role of baicalin in diabetic nephropathy treatment and the regulation of SIRT1/AMPK/HNF4A pathway remains unclear and requires future studies. Therefore, we investigated the potential targets and pathways of GDC and its active ingredient in the treatment of diabetic nephropathy by network pharmacology.

A previous study has shown that two protein targets, HNF4A and SIRT1, were proved as effective binding sites with GDC active ingredients by SPR (surface plasmon resonance) [10]. Our network pharmacology results indicated that baicalin might improve diabetic nephropathy through SIRT1, HNF4A, JAK3, and HMGCR. Furthermore, the targets HNF4A and SIRT1 were closely related to the AMPK pathway. Compared with the control group, there were decreased expressions of SIRT1 and p-AMPK*α* and increased expression of HNF4A in the kidney tissue of db/db mice and MPC-5 cell line, and GDC and baicalin could reverse these expression changes. Consistently, multiple studies have proved the crucial function of SIRT1, AMPK, and HNF4A as cellular signals in regulating lipid metabolism in nephrocytes [12, 22], where we further verified key molecules of this pathway by western blot.

Lipid accumulation in kidneys led to dysfunction and loss of podocytes, which was a vital common pathway in diabetic nephropathy [23, 24]. NPHS2 (podocin), a key protein of the podocyte slit diaphragm [25], was reduced in the kidneys of patients with massive proteinuria [24]. Our study demonstrated that GDC and baicalin restored the downregulated expression of podocin in db/db mice. In podocytes, both GDC and baicalin treatment reversed the decrease of podocin in renal podocytes stimulated by high glucose in a dose-dependent manner. Therefore, this study provided ample evidence that GDC and baicalin had a protective effect on diabetic nephropathy.

Our results proved the new role of HNF4A mediated by SIRT1 and AMPK signaling pathways in the lipid metabolism of diabetic nephropathy. Compared with the control group, the expressions of SIRT1 and p-AMPK*α* were decreased, while the expression of HNF4A was increased in the kidney tissue of db/db mice and MPC-5 cell line. GDC and baicalin could reverse these expression changes. Similar results were observed when MPC-5 was treated with different concentrations of GDC and baicalin.

It was proved that SIRT1/AMPK pathway affected the lipid homeostasis of kidney in many studies [12, 26, 27]. The phosphorylation of AMPK and activation of SIRT1 ameliorated lipotoxicity in the kidney and prevented apoptosis and oxidative stress in DN [28]. Lipid droplets (LDs) were used to store triacylglycerol and promoted oxidative metabolism via SIRT1-associated pathway [29]. AMPK increased the intracellular NAD^+^ levels to promote SIRT1 expression [30]; reciprocally, SIRT1 activated AMPK signaling pathway to regulate intracellular lipid accumulation in podocytes [12]. This suggested that there was a positive feedback loop between AMPK and SIRT1.

Hepatocyte nuclear factor 4 (HNF4) family regulated the transcription of lipid-related genes by binding to fatty acids [31], of which HNF4A regulated the hepatic lipid homeostasis via transcriptional regulation of VLDL-related genes [32]. The HNF4A expression decreased by the increased phosphorylation of AMPK, such as AMPK activator metformin [33]. AMPK directly phosphorylated HNF4A and repressed its transcriptional activity [34]. In addition, through RNA-seq analysis, HNF4A and AMPK*α*2 showed the opposite mRNA expression [35]. Herein, we demonstrated that GDC and baicalin downregulated the expression of HNF4A in the kidneys of model group in vivo. Likewise, both GDC and baicalin treatment significantly downregulated the expression of HNF4A in renal podocytes stimulated by high glucose in vitro. Consistent with those of previous studies, our findings indicated that the activation of AMPK might decrease the expression of HNF4A. However, the relationship between upstream and downstream of SIRT1/AMPK/HNF4A has not been verified in our studies which requires further research to seek the intrinsic link of these proteins.

## 5. Conclusions

Taken together, dyslipidemia in diabetic nephropathy mice was ameliorated by the downregulation of HNF4A and activation of AMPK and SIRT1, which could be related to abnormal lipid metabolism in renal podocytes in diabetic nephropathy. GDC ameliorated the development of diabetic nephropathy by reduction of lipids accumulation in the kidney via SIRT1/AMPK/HNF4A pathway. In addition, baicalin might be an effective component of GDC in improving diabetic nephropathy. Our data further substantiated that GDC attenuated lipid accumulation in HG-induced podocytes via SIRT1/AMPK/HNF4A, thus playing a protective role in renal podocytes injury.

## Figures and Tables

**Table 1:** Plant species and the composition of GDC.

Chinese phonetic alphabet	Botanic family	Botanical nomenclature	Amount (g)
Shan Zhu Yu	Cornaceae	*Cornus officinalis* Sieb. et Zucc.	416.7
Shu Di Huang	Scrophulariaceae	*Rehmannia glutinosa* Libosch.	416.7
Huang Qi	Leguminosae	*Astragalus mongholicus* Bunge	416.7
Yi Mu Cao	Lamiaceae	*Leonurus japonicus* Houtt.	500.0
Huang Qin	Lamiaceae	*Scutellaria baicalensis* Georgi	333.3
Huai Mi	Leguminosae	*Styphnolobium japonicum* (L.) Schott	333.3
Jiang Can	Stiff silkworm	*Bombyx Batryticatus*	333.3
Yu Gan Zi	Phyllanthaceae	*Phyllanthus emblica* L.	366.7
Total amounts			3116.7

## Data Availability

The datasets used and/or analyzed during the current study are available from the corresponding author on reasonable request.

## Abbreviations

GDC: Gandi capsule
DN: Diabetic nephropathy
ESRD: End-stage renal disease
TCM: Traditional Chinese medicine
SPR: Surface plasmon resonance
AMPK: 5′AMP-activated protein kinase
HNF4A: Hepatocyte nuclear factor 4-alpha
SIRT1: Silent information regulator 1
VLDL: Very low-density lipoprotein
TG: Triglyceride
TC: Total cholesterol
LDL-C: Low-density lipoprotein-cholesterol
HDL-C: High-density lipoprotein-cholesterol
MPC-5: Murine podocyte cell line.

## Supplementary Materials

Supplementary Materials Figures S1: TLC identification of Radix Astragali. Figures S2: TLC identification of Scutellaria. Figures S3: content determination of Radix Astragali. Figures S4: content determination of Scutellaria.
