# Supplementary material for: Gandi Capsule Improved Podocyte Lipid Metabolism of Diabetic Nephropathy Mice through SIRT1/AMPK/HNF4A Pathway
Source: Oxid Med Cell Longev. 2022 Apr 18;2022:6275505. doi: 10.1155/2022/6275505 (PMC9038418; doi:10.1155/2022/6275505)
Supplement: Supplementary Materials — Figures S1: TLC identification of Radix Astragali. Figures S2: TLC identification of Scutellaria. Figures S3: content determination of Radix Astragali. Figures S4: content determination of Scutellaria. [file 6275505.f1.pdf]

1. Identification

1.1. TLC identification of Radix Astragali

According to the thin layer chromatography test (general rule 0502 of China Herbal Pharmacopeia (2020)), the test solution and control drug solution was siphoned 5 μl, reference solution 3 μl, which were pointed on the same silica gel G thin-layer plate (TLC Silica gel 60 50Glass plates 10*20cm, MERCK, Lot: HX73381626) respectively. chloroform: methanol: water (13:7:2) as developing agent. Then developed, taken out and dried. 10% sulfuric acid ethanol solution (V / V) was sprayed and heated at 105 ℃ until the spots are clear. In the chromatogram of the test sample, spots of the same color were found on the corresponding positions of the reference material and the chromatogram of the reference material.

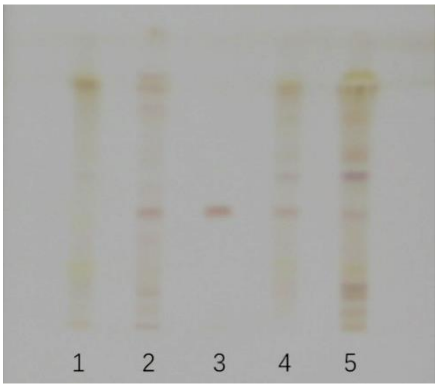

Figure 1 1: negative control, 2: Radix Astragali, 3: Astragaloside IV, 4: test solution, 5: GDC capsule

1.2. TLC identification of scutellaria

Methanol (30ml) was added into the GDC mixture (3g), and the mixture was heated and refluxed in a water bath for 30min, then the residue was dissolved with methanol. In addition, baicalin reference substance was added with methanol to make a solution containing 1mg per 1ml as the reference substance solution. According to the thin layer chromatography test (general rule 0502), the solution was siphoned 2 μl, and was developed with ethyl acetate butanone formic acid water (5:3:1:1) as developing agent, then taken out, dried and sprayed with 5% ferric chloride ethanol solution. In the chromatogram of the test sample, spots of the same color were found at the corresponding positions of the chromatogram of the reference sample.

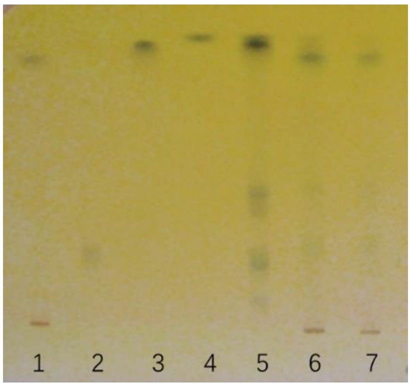

Figure 2 1: negative control, 2: Baicalin reference substance, 3: Baicalein reference substance, 4: Wogonin reference substance, 5: Control medicinal solution, 6: GDC capsule,

7: test solution

2. Content determination

2.1. Radix Astragali

Astragaloside IV was determined by HPLC (general rule 0512 of China Herbal Pharmacopeia (2020)). Octadecylsilane bonded silica gel was used as the filler in chromatographic conditions and system applicability test; The mobile phase was acetonitrile water (32: 68); Evaporative light scattering detector. The number of theoretical plates should not be less than 4000 according to astragaloside IV peak.

Preparation of the reference solution: Appropriate amount of astragaloside IV was accurately weighed it, 80% methanol was added to make a solution containing 0.5mg per 1ml.

Preparation of test solution: Appropriate amount of powder (passing through No.4 sieve), was weighed it precisely. 50 ml 80% methanol solution containing 4% concentrated ammonia test solution was added into a conical flask with stopper, then heated and refluxed for 1 hour, which was evaporated to dryness, dissolved with 80% methanol, and transfered to a 5ml volumetric flask.

5 μl the standard solution and 10 μl test solution was precisely siphoned, and injected into liquid chromatography. external standard two-point method was used to analyzed the results.

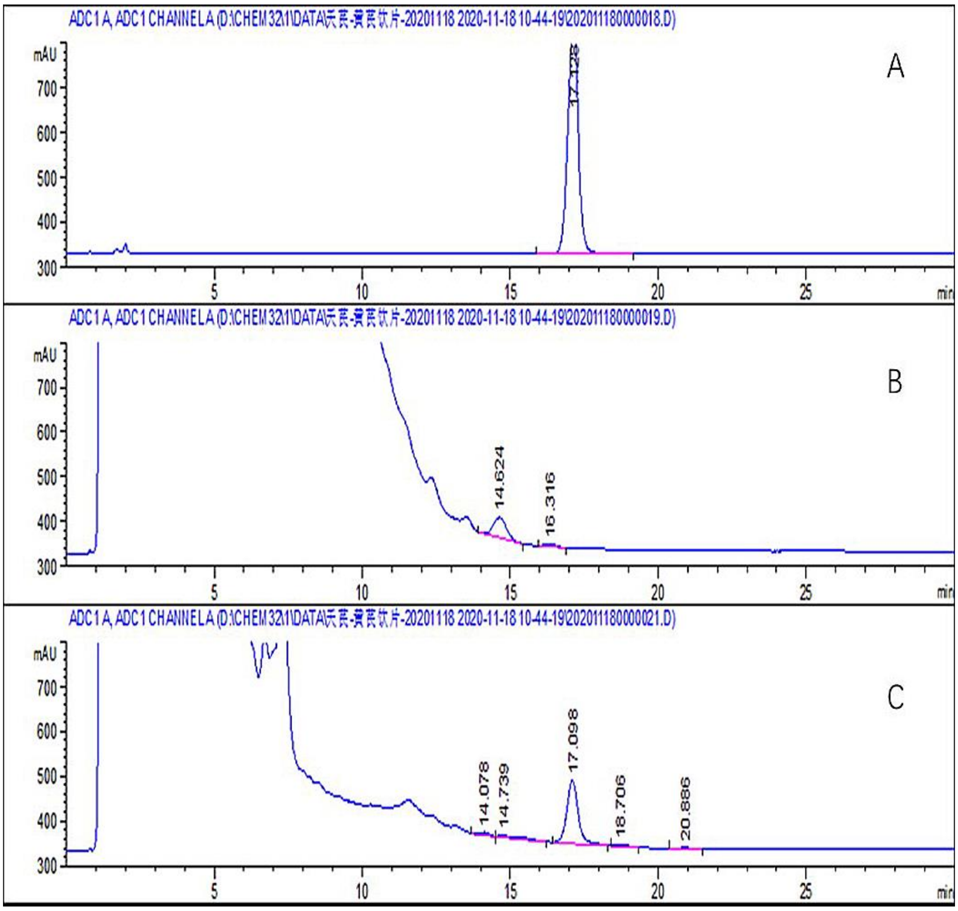

Figure 3 A: reference solution of astragaloside IV, B: negative control, C: test solution

2.2. scutellaria

Octadecylsilane bonded silica gel was used as the filler in chromatographic conditions and

system applicability test. The mobile phase was methanol-0.4% phosphoric acid (43:57). The detection wavelength was 280nm. The number of theoretical plates should not be less than 2500 according to baicalin peak.

Preparation of reference solution: Appropriate amount of baicalin reference was accurately weighed, and methanol was added to make a solution containing 0.1mg per 1ml.

Preparation of test solution: GDC mixture was weighted about 0.1g precisely, dissolved with 70% ethanol, and transferred into a 50ml volumetric flask. Ultrasonic treatment (power 250W, frequency 40KHz) for 30 minutes. 70% ethanol was added to the scale.

The standard solution and the test solution were precisely aspirated for 10 μl, and injected into liquid chromatography. external standard two-point method was used to analyzed the results.

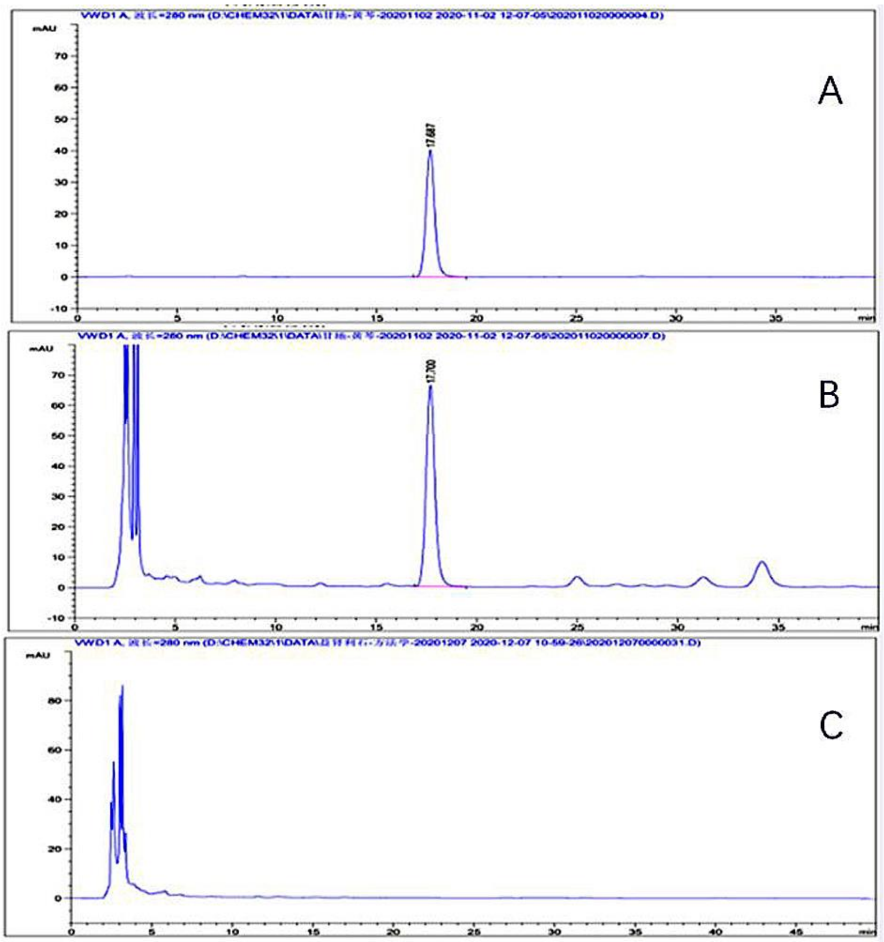

Figure 4 A: reference solution of baicalin, B: test solution, C: negative control
